# Modelling Nutritional Requirements of Growing Pigs from Local Breeds Using InraPorc

**DOI:** 10.3390/ani9040169

**Published:** 2019-04-16

**Authors:** Ludovic Brossard, Rosa Nieto, Rui Charneca, José Pedro Araujo, Carolina Pugliese, Čedomir Radović, Marjeta Čandek-Potokar

**Affiliations:** 1 PEGASE, INRA, Agrocampus-Ouest, 35590 Saint-Gilles, France; 2 EEZ, Spanish National Research Council (CSIC), Profesor Albareda s/n, 18008 Granada, Spain; rosa.nieto@eez.csic.es; 3 ICAAM—Instituto de Ciências Agrárias e Ambientais Mediterrânicas, Universidade de Évora, Pólo da Mitra, Ap. 94, 7006-554 Évora, Portugal; rmcc@uevora.pt; 4 Instituto Politécnico de Viana do Castelo (IPVC)—Centro de Investigação de Montanha (CIMO), Praça General Barbosa, 4900 Viana de Castelo, Portugal; pedropi@esa.ipvc.pt; 5 Dip.Scienze e Tecnologie Agrarie, University of Florence, Alimentari, Ambientali e Forestali (DAGRI), Via delle Cascine 5, 50144 Firenze, Italy; carolina.pugliese@unifi.it; 6 IAH-Institute for Animal Husbandry, Autoput za Zagreb 16, 11080 Belgrade-Zemun, Serbia; cedomirradovic.izs@gmail.com; 7 KIS-Agricultural Institute of Slovenia, Hacquetova ul. 17, 1000 Ljubljana, Slovenia; meta.candek-potokar@kis.si

**Keywords:** local breeds, growing pigs, modelling, nutrition, requirements

## Abstract

**Simple Summary:**

Unlike with conventional pig breeds, knowledge on growth and performance, and even more so on nutritional requirements, is very limited for local breeds. Using a modelling approach based on a growth model and data from literature or experiments from H2020 European Union project TREASURE, we determined the growth characteristics and nutritional requirements of nine local breeds (Alentejana, Basque, Bísara, Apulo Calabrese, Cinta Senese, Iberian, Krškopolje pig, Mangalitsa, and Moravka). Our results confirmed the lower growth potential of local pig breeds compared to conventional pig breeds. Moreover, a larger proportion of ingested and retained energy is dedicated to lipid deposition in local pig breeds, explaining the higher fat composition of the carcasses of these breeds. Our study provided initial insights into the nutritional requirements (such as amino-acids) of local pig breeds, providing a first step towards defining feeding strategies better adapted to the characteristics of these breeds.

**Abstract:**

Models such as InraPorc enable the growth of pigs to be simulated and their nutrient requirements to be determined. However, so far, these models have not been applied to local breeds. We used InraPorc to determine the nutrient requirements of growing pigs from local breeds (H2020 European Union project TREASURE). Data on feed composition, allowance and intake, and body weight (BW) were obtained from literature reports or experiments conducted within the project. Data were used in InraPorc to calibrate 16 growth and intake profiles from nine breeds (Alentejana, Basque, Bísara, Apulo Calabrese, Cinta Senese, Iberian, Krškopolje pig, Mangalitsa, and Moravka), with one to three profiles per breed depending on the experimental conditions or data source. On the 40–100 kg BW range, mean protein deposition (PDm) was low for all breeds (below 116 g/d vs. over 130 g/d in conventional breeds). The age of pigs at 40 kg BW (110 to 206 days) denoted different types of feeding management in addition to genetic differences. The PDm and the lysine requirements were the highest in breeds with the highest average daily gain. In all breeds, a small proportion of total body energy retention was dedicated to protein, with the greatest proportion of energy retention in the form of lipids. Despite some methodological limitations, this study provides initial insights into the nutrient requirements of some local breeds.

## 1. Introduction

Modelling approaches have been developed since the 1970s to predict the response of growing pigs to nutrient supply and to simulate their performance (growth, feed intake, lipid and protein deposition, etc.) [1,2]. The models have been largely applied to conventional breeds. Indeed, performance data and information on diets are often necessary to parameterize the models or compare simulation results to observed performance. Additionally, to obtain reference parameters for a genotype, the modelling approach requires data obtained in conditions that allow full expression of animal potential for feed intake and growth. For conventional breeds, such data, for different stages or for the entire growth period, are numerous and easy to obtain from field trials or previously published studies. Experimental results on nutrient requirements are also abundant for modern pig genotypes. Some models have been used as decision tools to synthetize data on requirements, determine nutrient requirements, and identify adapted feeding strategies depending on the genotype (i.e., InraPorc) [3,4].

Current knowledge on the growth and performance of local pig breeds is very limited. There have been few studies showing the specific metabolic characteristics of local breeds [5] and their lower rates of growth and lean-tissue deposition [6] compared to conventional, genetically improved pig breeds. This particular metabolic profile implies that they also have specific nutritional requirements. For autochthonous or local pig breeds, the data on their growth, feed ingestion capacity, and nutrient requirements are very scarce and of limited adequacy for modelling, except for more developed breeds, such as Iberian pigs. Although optimum dietary protein/energy ratios for growing and fattening Iberian pigs have been studied [7], additional studies on different productive phases and on other local pig breeds are needed in order to acquire knowledge of their nutritional requirements prior to optimizing growth and performance of autochthonous pig breeds. Furthermore, to our knowledge, modelling approaches have not been applied yet to pigs from local breeds and/or reared in non-conventional systems. Indeed, studies on local breeds are often made in very diverse conditions of feeding and management, many of them reflecting the practical use. Only a small number of the studies performed on these breeds offer adequate data that allow for the evaluation of each breed’s potential for growth.

The present study aimed to apply a modelling approach with InraPorc model to determine the nutrient requirements of growing pigs from local breeds in the H2020 European Union project TREASURE.

## 2. Materials and Methods

### 2.1. Data Requirements for Modelling Study

InraPorc is a model and a decision support tool developed to evaluate the impact of nutritional strategies on performance and nutrient utilization in pigs. Calculation of nutrient requirements for growing pigs with InraPorc requires defining an animal profile describing the potential for growth and feed intake of the studied pigs. In the model, *ad libitum* daily feed intake (DFI) in MJ net energy (NE) was modelled as a gamma function of body weight (BW) expressing DFI in multiples of NE intake above maintenance with two parameters ‘a’ (dimensionless) and ‘b’ (per kg BW). To let users have an easier understanding of DFI parameters, ‘a’ and ‘b’ can be transformed to DFI at 50 kg BW and at 100 kg BW (respectively NE_50_ and NE_100_, in MJ/NE). Growth is described through protein deposition (PD) modelled by a Gompertz function that was parameterized with three parameters: BW at the considered initial age (BWinit, kg), mean protein deposition between initial age and considered final BW (PDm, g/day), and the shape parameter of the Gompertz function (B_Gompertz_, per day) describing the precocity of PD and thus the growth. For this B_Gompertz_ parameter, a profile with a high value of B_Gompertz_ indicates a high intensity of growth and protein deposition (fast increase in growth rate and protein deposition) in the first stage of growth period and a fast decrease in these rates during the finishing period. Conversely, a low value of B_Gompertz_ indicates a less intensive but more persistent increase of growth rate and protein deposition during the growing period with a smaller decrease of this rate when approaching the maturity. To summarize, a profile describes the potential of a considered pig using five parameters, two for DFI and three for growth. 

These five parameters can be obtained through a calibration process requiring real data on BW (at least at the beginning, middle, and end of the considered growth period), mean DFI (at least subdividing the considered growth period in two parts), and information (raw materials and/or nutrient composition) on feed offered to pigs during the considered period [8]. An additional condition is that the required data on growth and feed intake must be obtained under an *ad libitum* feed allowance during a major part of the period, in particular at the initial stage of growth. 

### 2.2. Data Collection

Growth modelling with the InraPorc model was performed for local pig breeds of the TREASURE project ([9]; www.treasure.kis.si) for which the appropriate data (body weight measured at least at three time points, and feed intake recorded throughout the monitored period) was available. The data was obtained either from field trials conducted in the TREASURE project (*n* = 5) or extracted from already published studies (*n* = 5). Feed composition, feed sequence plan, feed allowance, growth, and feed intake data were collected for each trial. In total, data was obtained for 16 trials for the following breeds: Portuguese breeds Alentejana and Bísara, French breed Basque, Italian breeds Apulo Calabrese and Cinta Senese, Spanish breed Iberian, Slovenian breed Krškopolje pig, and Serbian breeds Mangalitsa (Swallow-Belly type) and Moravka. The principal characteristics of the selected studies and trials are indicated below. Feed characteristics are given expressed on an as-fed basis. For more details, see Table 1 and Appendix A. This work did not involve any experimentation with animals that would demand ethical protocols according to Directive 2010/63/EU (2010). It was undertaken using the data from previously published and/or recorded data.

#### 2.2.1. Alentejana

For the Alentejana breed, we used data published in the doctoral thesis of Freitas [10], in which two trials were conducted using, as stated, approximately *ad libitum* feeding (Table A1). From the first trial data on 22 pigs (half males, half females, both castrated) between 41.5 and 96.3 kg live body weight (BW) was used. During the whole trial, pigs received the same feed mixture (9.14 MJ NE/kg, 15.7% protein). Regarding the second trial, data from 39 pigs (until 60 kg BW) or 13 pigs (60 to 95 kg) from 35.3 to 95.1 kg BW was used. Pigs were also fed the same feed mixture during the whole trial (8.51 MJ NE/kg, 15.2% protein). The trials lasted 118 and 136 days, respectively. 

#### 2.2.2. Basque

Data published by Lebret et al. [11] was used for the Basque breed (Table A2). The article reported data for two experimental groups of castrated males held in two different production systems: conventional (slatted floor, no outdoor access, 1.0 m²/pig) and alternative (indoor straw bedding and free permanent access to an outdoor area on concrete floor, 2.4 m^2^/pig). For the first group, the data refers to pigs between 34.8 kg and 139.9 kg BW, and for the second group, between 35.3 and 146.3 kg. Pigs received two different diets adapted to growing stage (9.59 MJ NE/kg, 18.0% protein and 8.96 MJ NE/kg, 14.7% protein). The trials lasted 214 and 206 days, respectively. Pigs were fed *ad libitum* until 75 kg BW. 

#### 2.2.3. Bísara

Data reported in a study of Santos e Silva et al. [12] and data from the trial performed in project TREASURE were used (Table A3). In the first study, the same feed mixture (9.51 MJ NE/kg, 15.0% protein) was used for the whole trial duration (209 days, from 29.8 to 144.3 kg BW). Entire male pigs, castrated male pigs, and female pigs were used. In the second trial, data on pigs (*n* = 10) in a confinement system, of two sexes (castrated males and females) between 25.3 and 115.8 kg BW (168 days) was used for modelling. A starter diet containing 9.75 MJ NE/kg, 16.7% protein was distributed for 28 days. Then, a growth diet containing 9.43 MJ NE/kg, 15.3% protein was distributed for 140 days. During the period on the growth diet, pigs were additionally given ground corn (9.90 MJ NE/kg, 8.6% protein) as follows: 0.3 kg for the first 28 days, then 0.4 kg for 42 days, and 0.6 kg for the last 70 days.

#### 2.2.4. Apulo Calabrese

For the Apulo Calabrese breed, the data published by Rossi et al. [13] was used (Table A4). In the mentioned study, a two-phase feeding (i.e., different feed mixtures for pigs between app. 30–70 kg and 70–130 kg) was reported. Two diets with soy (9.22 MJ NE/kg, 15.5% protein and 9.70 MJ NE/kg, 14.2% protein) or without soy (9.34 MJ NE/kg, 15.4% protein; 9.69 MJ NE/kg, 14.2% protein) were distributed to pigs between 33.8 and 135.2 kg BW, and 40.2 kg to 139.1 kg BW, respectively. The trial lasted 142 days and was conducted with 72 pigs (36 pigs per diet; sex was not specified). 

#### 2.2.5. Cinta Senese

Data from the experiment conducted in project TREASURE was used for modelling (Table A5). Growth and feed intake data for a group of eleven pigs (castrated males) between 38.0 kg and 143.4 kg BW were used. Pigs were fed two diets: until app. 85 kg, a diet containing 9.64 MJ NE/kg, 12.4% protein, and thereafter a diet with 9.71 MJ NE/kg, 10.33% protein. The duration of the trial was 246 days. Pigs were weighed every 21 days and provided a quantity of feed corresponding to 3% of their BW. 

#### 2.2.6. Iberian

Data was collected from two published studies. Ayuso et al. [14] studied the growth of castrated pigs (*n* = 20–29) in the BW interval 16 to 160 kg (for 273 days) using three different diets according to growth stage (10 MJ NE/kg, 17.8% protein; 9.50 MJ NE/kg, 15.8% protein; and 10.40 MJ NE/kg, 13.5% protein; Table A6). Barea et al. [15] studied the growth of castrated males from 16 to 114 kg BW, and pigs received the same diet throughout the study (Table A7). Two groups of pigs differing in dietary protein level were analyzed separately: one group (*n* = 4–8) received a diet with 9.23 MJ NE/kg, 12.6% protein, and the other (*n* = 4–8) a diet with 9.08 MJ ME/kg, 16.6% protein. Trials lasted 155 and 174 days respectively.

#### 2.2.7. Krškopolje Pig

Data was collected in project TREASURE for two group of pigs (castrated males), the first one being held in a conventional (indoor) production system (*n* = 12) and the second one being reared respecting the standards of ecological farming and ecological feed mixture ingredients (*n* = 12) (Table A8). Two-phase feeding was used. In the conventional system, the pigs were monitored between 36.2 kg and 120.4 kg BW and received a diet with 8.52 MJ NE/kg, 15.3% protein for 28 days and thereafter a diet with 8.82 MJ NE/kg, 14.0% protein. In the ecological production system, the pigs were monitored between 35.0 kg and 124.3 kg BW and received a diet with 8.69 MJ NE/kg, 15.0% protein for 28 days and thereafter a diet with 8.83 MJ NE/kg, 14.0% protein. Pigs in both groups were fed *ad libitum* until about 70 kg. The duration of the trial was 115 days. 

#### 2.2.8. Mangalitsa and Moravka

For the Mangalitsa and Moravka breeds, the data was collected in project TREASURE (Table A9). The trial was performed with castrated males (*n* = 12 and *n* = 10 for Mangalitsa and Moravka breeds, respectively). Pigs were held in two pens of 60 m^2^ with access to a large outdoor area (130 m^2^). They received two diets: the first 82 days of trial (until app. 60 kg BW for Mangalitsa breed and 70 kg BW for Moravka breed) a diet with 9.98 MJ NE/kg, 13.7% protein, and the last 116 days a diet with 10.0 MJ NE/kg, 14.4% protein. 

### 2.3. Growth Modelling

Collected information consisting of BW and DFI data, as well as feed composition, feed allowance, and feed sequence plan (which diet offered at each growth phase) was entered in InraPorc separately for each trial. The nutrient composition of the feed was recalculated using raw material composition when available and corrected when analyzed composition was available. Profiles were then calibrated and 16 growth profiles were obtained. Simulations were performed for each profile using corresponding feed and feed sequence, and an *ad libitum* feed allowance to enable the calculation of performance, energy and lysine repartition depending on growth potential, and standardized ileal digestible (SID) lysine requirements. The initial and final BW in the trials ranged between 15.7 and 40.9 kg and between 95.5 and 159.5 kg, respectively. Simulations with InraPorc cannot be performed from a lower BW than the observed one. Simulations can be performed according to a higher or lower final BW than the BW that was actually observed. However, parametrization is dependent upon available BW data and two profiles based on the same BW data, except the final BW (for instance, 100 kg and 140 kg), can have different B_Gompertz_ (shape of the curve) and PDm. Consequently, extrapolating performance to a higher BW than the BW that was actually observed can induce bias in growth performance estimation. Therefore, performance was calculated within the range of 40–100 kg BW that was common to all trials.

## 3. Results and Discussion

InraPorc parameters and simulation results between 40 and 100 kg BW for the 16 profiles are presented in Table 2. 

Ages at 40 kg and 100 kg BW ranged between 110 and 206 days, and between 195 and 323 days, respectively. This difference between studied breeds is also revealed by profile parameters and may stem from genetic differences or in feeding management (or both) among breeds before the monitored growth period. It should be noted that in the InraPorc model, potential PD is driven by state (i.e., current protein content in body) and not by age [3]. This means that in cases where two animals had the same BW at the start, even if they have very different ages, the same PD can be observed if the current protein content in body is close. As such, the period before the monitored growth period can influence the modelling results, for instance, by impacting the protein content in body at the start and thus potential PD in following period.

For the interval 40–100 kg BW, the PDm ranged between 41 and 105 g/d. This can be considered rather low when compared to values reported for conventional, genetically improved breeds. Applying the same procedure as in the present study, Vautier et al. [8] obtained profiles from pigs of nine commercial pure or synthetic lines. In their results, the PDm for a 30–110 kg BW interval, quite comparable to the 40–100 kg BW interval in the present study, ranged between 130 and 166 g/day. Differences observed in PDm among the studied local breeds could reflect genetic differences. Alentejana, Cinta Senese, Mangalitsa, and Moravka presented PDm lower than 55 g/d, whereas others breeds had PDm higher than 65 g/d. Data on protein deposition are scarce for local breeds, although in Iberian pigs, studies to determine protein deposition in different growing phases have been reported. Nieto et al. [16] reported a PD of 74 g/day and Barea et al. [6] reported a PD of 71 g/day for Iberian pigs followed, respectively, from 15 to 50 kg BW and from 50 to 100 kg BW and fed adequate protein diets to each phase at a feed intake level close to *ad libitum*. These values are close to those obtained in this simulation study.

**Figure 1:**
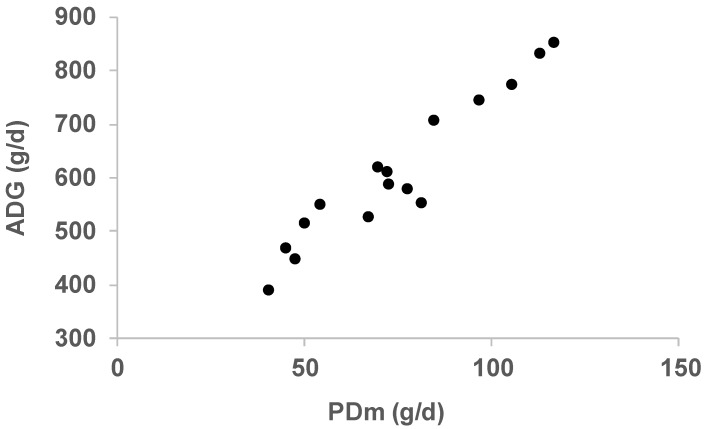
Relationship between mean protein deposition (PDm, g/d) and average daily gain (ADG, g/d) for nine local breeds during the period 40–100 kg BW (see correspondence between points’ values and InraPorc profiles in Table 2).

The average daily gain (ADG) ranged between 389 to 854 g/d. Even if the relation between PDm and ADG is not strictly linear, breeds with higher PDm presented higher ADG, and conversely, breeds with lower PDm presented lower ADG (Figure 1). Local breeds are known to have lower growth potential, and thus lower protein deposition rates, than conventional breeds that can reach ADG values higher than 1000 g/d in optimal conditions under intensive rearing systems. Candek-Potokar et al. [17] have reviewed the performance characteristics of local pig breeds, including those of the present study, for different growth periods. For the period 30–100 kg BW, quite comparable to the BW range chosen in our study, the pooled average ADG found for the breeds selected was 477 g/d vs. 590 g/d obtained in the present study. Some profiles fitted well with data from Candek-Potokar et al. [17]. For instance, those authors reported an ADG of 483 g/d for the Alentejana breed, 508 g/d for the Moravka breed, and 412 g/d for the Cinta Senese breed vs. 459 g/d, 550 g/d, and 389 g/d, respectively, in the present study. Conversely, larger differences were observed for the Krškopolje pig (580 g/d vs. 854 g/d in the present study) or the Apulo Calabrese breed (418 vs. 760 g/d in the present study), for instance. This can be partly explained by the type of data used for calibration and by *ad libitum* simulation compared to field practices.

In the studied local breeds, B_Gompertz_ ranged between 0.0024 and 0.0183/d. As for growth potential, local breeds presented lower values than conventional breeds. Vautier et al. [8] reported B_Gompertz_ ranging between 0.0129 and 0.0256/day for conventional breeds. This indicates that local breeds have generally less intensive but more persistent growth than conventional breeds. However, the range of B_Gompertz_ values also reflects a large difference between the studied local breeds.

**Figure 2:**
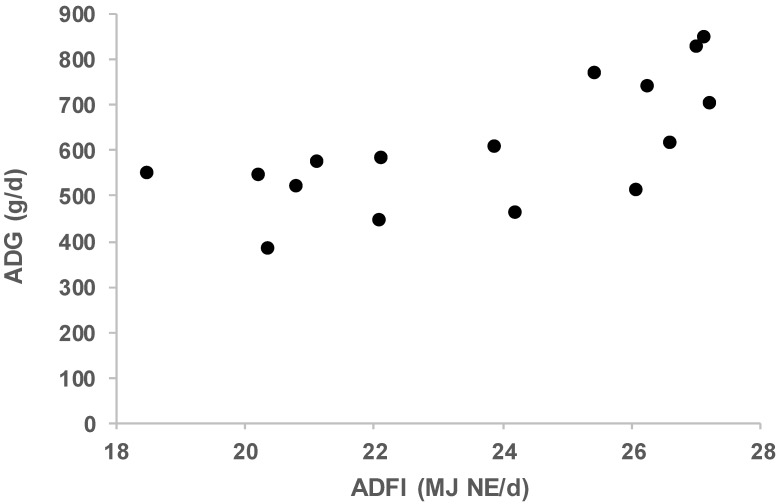
Relationship between average daily feed intake (ADFI, MJ NE/d) and average daily gain (ADG, g/d) for nine local breeds during the period 40–100 kg BW (see correspondence between points’ values and InraPorc profiles in Table 2).

The ADFI, NE_50_, and NE_100_ values ranged between 18.5 and 27.2 MJ NE/d, 15.7 and 24 MJ NE/d, and 19.3 and 35.2 MJ NE/d, respectively. The breeds with higher ADGs presented generally higher ADFI even if the relationship was not strict (Figure 2). For conventional breeds, Vautier et al. [8] reported NE_50_ values ranging between 18.6 and 24.0 MJ NE/d and for NE100 ranging between 23.7 and 31.2 MJ NE/d. The difference between conventional and local breeds is not so clear here. In the present study, we collected data in feeding conditions that were supposed to be *ad libitum*. Assuming a ratio NE/metabolizable energy (ME) of 0.74 and the NRC (National Research Council) value for ME for maintenance (448 kJ ME per kg BW0.75; [4]), and using feed allowance indicated in Table 1, it can be estimated then that feed allowance ranged in our data from 2.23 (Iberian study 1) to 3.32 (Mangalitsa and Moravka) times ME for maintenance. In reference to the assumption that voluntary DFI equals approximately 3 to 4 times the ME requirements for maintenance, some breeds could have been somewhat below *ad libitum* conditions.

**Figure 3:**
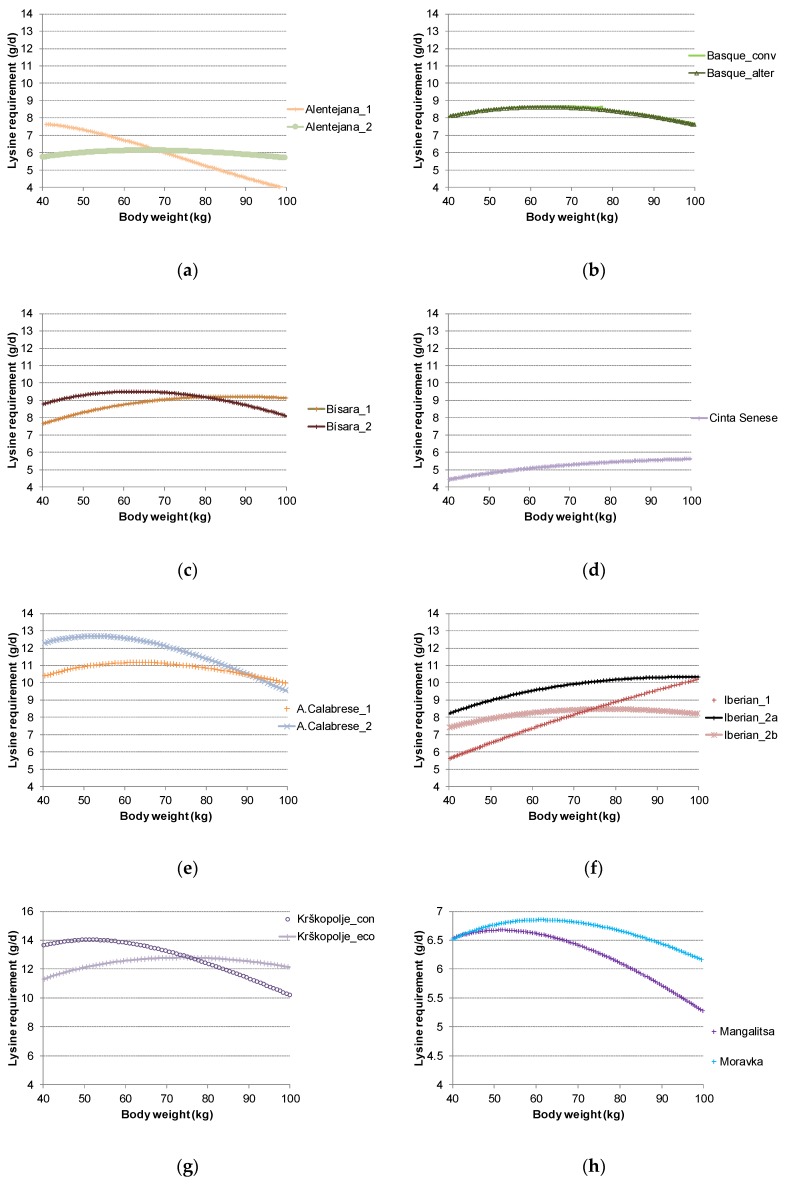
Standardized ileal digestible lysine requirement (g/d) for nine local breeds depending on body weight (kg) during the period 40–100 kg BW: (**a**) Alentejana; (**b**) Basque; (**c**) Bísara; (**d**) Cinta Senese; (**e**) Apulo Calabrese; (**f**) Iberian; (**g**) Krškopolje; (**h**) Mangalitsa and Moravka.

The average SID lysine requirement between 40 and 100 kg BW ranged between 5.2 to 12.8 g/d. In the present study, the SID lysine requirements accounted for between 25% and 75% of dietary supplies, indicating that the studied breeds were not limited in lysine supply in this simulation. There is a clear and logical relationship between lysine requirement and PDm or ADG, with the breeds with higher PDm or ADG presenting higher requirements. Indeed, the SID lysine requirement is mainly due to protein deposition. In addition to average values, the InraPorc model allows the kinetics of evolution to be described for different criteria. The evolution of SID lysine requirements for the obtained profiles depending on BW is presented in Figure 3. A diversity of kinetics is observed beyond the range of mean values, also linked to B_Gompertz_ values, which are indicators of the shape of growth rate and PD curves, as explained above. For instance, the simulations for the two profiles of the Alentejana breed resulted in similar SID lysine requirements but different kinetics. For the Alentejana_1 profile, the kinetic indicates a decrease of requirements in the 40–100 kg BW range, whereas the kinetic for the Alentejana_2 profile is flatter. This can be linked to the higher B_Gompertz_ value for the Alentejana_1 profile compared to the Alentejana_2 profile. More generally, in the present study, the profiles with higher B_Gompertz_ values presented more pronounced curves for SID lysine requirement kinetics. 

**Figure 4:**
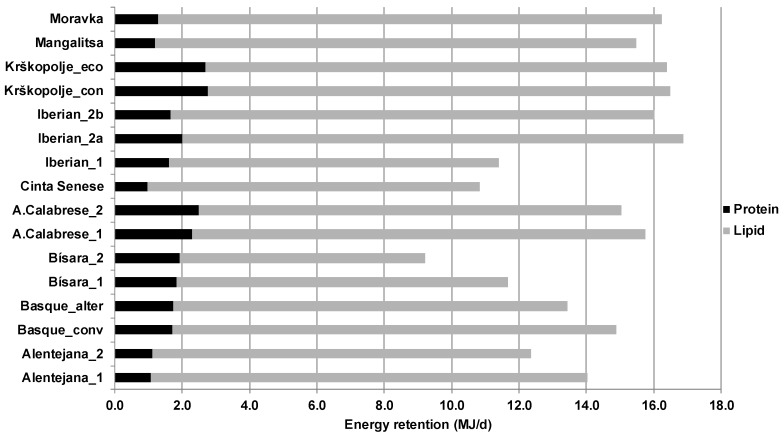
Retained energy in protein or lipid (MJ/d) for nine local breeds during the period 40–100 kg BW.

The energy retained in protein and lipid ranged between 0.97 and 2.77 MJ/d and 7.28 and 14.95 MJ/d, respectively (Figure 4). The total energy retention ranged between 9.22 and 16.88 MJ/d. In all breeds and profiles, a low proportion (8% to 21%) of total energy retention was directed towards protein, with the remaining proportion being dedicated to lipid deposition. In InraPorc, ingested energy is modeled as being used first for maintenance functions (including physical activity), with the remaining energy being used for protein deposition and then for lipid deposition. As such, lipid deposition is considered as an energy sink, as often the case for modelling approaches on pig growth [12]. Energy deposition in the studied breeds, presenting low PD, is then oriented mainly to lipid deposition, which is taken into account in the model. This reflects also the observations that pigs from local breeds are considered to be fatty pigs, or at least fatter than conventional breeds [18]. As indicated in the review of Candek-Potokar et al. [18], the average lean meat percentage for the local breeds included in the present study was situated between 32.9% and 48.4%, and the average loin eye area was between 18.1 to 36.3 cm^2^, which demonstrates low muscular development. With lower PDm than conventional breeds but comparable NE_50_ and NE_100_, a larger part of ingested and retained energy is dedicated to lipid deposition, explaining the higher fat composition of the carcasses of these breeds [18].

The InraPorc profiles obtained in the present study reflect the genetic differences among the breeds concerning NE intake, growth capacity, and precocity of growth. For breeds where several profiles have been calibrated, profiles of the same breeds are more or less comparable. For instance, both of the Alentejana profiles have quite comparable parameters, except for B_Gompertz_. The differences among Iberian profiles are more pronounced on some parameters, such as B_Gompertz_ between data source, or even for the same source, depending on the diet composition parameters (e.g., ADG). Calibration of profiles in InraPorc depends on the availability of data fitting with calibration process. InraPorc parameters in a profile representing a specific type of pigs reveal genetic differences but also reflect rearing conditions in which the data used for profile calibration were obtained [3]. Indeed, even if pigs are fed *ad libitum*, rearing conditions such as ambient temperature, diet composition, space allowance, or health status can influence the expression of growth potential through the effect on feed intake level or nutrient use. For instance, energy requirements for maintenance or activity can increase depending on space allowance or temperature. This can imply lower availability for protein deposition. Moreover, this can affect the level of observed performances without deviating growth and feed intake kinetics from a normal shape. Thus, it is important to emphasize that an animal profile in InraPorc reflects a phenotype (i.e., the potential of a genotype reared in a specific environment and submitted to management practices). For breeds with several profiles, we could observe that the conditions of data collection clearly influenced the calibration results. Some approximations were needed for calibration in the present study (e.g., feed allowance was supposed to be *ad libitum* following information given in some studies or recalculated nutrient composition of some diets when there was an uncertainty on real composition). These different elements can partly explain differences in profile parameters for the same breed.

## 4. Conclusions

Despite some methodological limitations due to the availability of data fitting with the needs of the calibration process, the present study provides initial insights into the modelled growth characteristics of some local breeds. Indeed, to our knowledge, this methodology has never been previously applied for these breeds. Parameters such as precocity (reflecting the shape of growth kinetic), PDm, or intake curve characteristics were obtained and have been used to determine nutrient requirements (here for SID lysine). These elements are currently scarce for most of the local breeds, even experimentally. Such breeds are often studied in field conditions that impair the definition of growth and intake potential or nutrient use. Important differences between local breeds were determined regarding growth potential, reflecting genetic differences but likely also differences in management during the lactation and post-weaning periods. These results are preliminary and have to be completed and refined using more data, for instance, with a better estimation of feed intake and feed composition, especially for non-usual feed such as forage. However, they can be used to help improve their feeding management by defining feeding strategies more adapted to the characteristics of local pig breeds, including better fitting of feed allowance or feed composition to the growth potentials of these pigs. This could allow for better optimization of feeding, while also reducing feed costs, nitrogen emissions, and controlling meat quality. Consequently, this could help to improve the sustainability and development of local pig breeds.

## Figures and Tables

**Table 1:** Summary of studies selected for modelling study on local breeds (diet composition and feed intake expressed on an as-fed basis). BW: body weight; NE: net energy.

Breed ^1^	Data Source	No. of	Days	Initial	Final	Diet(s) Composition		Feed Intake,
		Pigs	on Trial	BW, kg	BW, kg	Energy, MJ NE/kg	Protein, %	kg/Pig/d
Alentejana_1	[10]	28–34	118	41.5	96.3	9.14	15.7	2.59
Alentejana_2	[10]	13–39	136	35.3	95.1	8.51	15.2	2.45
Basque_conv	[11]	20	214	34.8	139.9	9.59	18.0	2.39
						8.96	14.7	
Basque_alter	[11]	20	206	35.3	146.3	9.59	18.0	2.68
						8.96	14.7	
Bísara_1	[12]	24	209	29.8	144.3	9.51	15.0	2.34
Bísara_2	TREASURE project	20	168	25.4	115.8	9.75	16.7	1.5 kg + corn (0.0–0.6 kg)
						9.43	15.3	
						Corn: 9.90	8.6	
A.Calabrese_1	[13]	36	142	40.2	139.1	9.34	15.4	3.14
						9.69	14.2	
A.Calabrese_2	[13]	36	142	33.8	135.2	9.22	15.5	3.00
						9.70	14.2	
Cinta Senese	TREASURE project	11	246	38.0	143.4	9.64	12.4	2.48
						9.71	10.3	
Iberian_1	[14]	20–29	273	16.1	159.5	10.0	17.8	2.10
						9.5	15.8	
						10.4	13.5	
Iberian_2a	[15]	6–8	155	15.7	114	9.23	12.6	2.54
Iberian_2b	[15]	6–8	174	16.0	114	9.08	16.6	2.55
Krškopolje_con	TREASURE project	12	115	36.2	120.4	8.52	15.3	3.23
						8.82	14.0	
Krškopolje_eco	TREASURE project	12	115	35.0	124.3	8.69	15.0	3.20
						8.83	14.0	
Mangalitsa	TREASURE project	12	198	24.3	115.5	9.98	13.7	2.64
						10.00	14.4	
Moravka	TREASURE project	10–12	198	29.9	131.3	9.98	13.7	2.94
						10.00	14.4	

^1^ The numbers (1, 2, 2a, 2b) or indications (conv: conventional; alter: alternative; con: conventional; eco: ecological) following the names of the breeds refer to different studies (see details in the text and in Appendix A).

**Table 2:** InraPorc parameters of profiles and simulation results between 40 and 100 kg BW for nine local breeds ^1^.

Profile ^2^	InraPorc Parameters						Performance 40–100 kg BW		
	NE_50_, MJ NE	NE_100_, MJ NE	B_Gompertz_, /d	PDm, g/d	Age at 40 kg, d	Age at 100 kg, d	ADG, g/d	ADFI, MJ NE/d	SID Lys. req., g/d
Alentejana_1	19.0	29.4	0.0143	45	154	281	468	24.2	5.8
Alentejana_2	17.9	27.4	0.0070	47	148	282	449	22.1	6.0
Basque_conv	21.5	22.2	0.0096	72	114	212	613	23.9	8.4
Basque_alter	23.6	27.9	0.0096	72	117	219	588	22.1	8.3
Bísara_1	18.1	24.5	0.0073	77	127	230	580	21.1	8.8
Bísara_2	17.2	19.3	0.0100	81	121	229	554	18.5	9.1
A.Calabrese_1	20.8	33.3	0.0123	97	160	241	746	26.2	10.8
A.Calabrese_2	19.7	32.7	0.0163	105	150	228	774	25.4	11.7
Cinta Senese	15.7	27.8	0.0041	41	125	280	389	20.3	5.2
Iberian_1	16.3	28.9	0.0024	67	143	258	526	20.8	7.8
Iberian_2a	24.0	30.4	0.0079	84	110	195	707	27.2	9.7
Iberian_2b	23.6	29.1	0.0083	69	113	210	619	26.6	8.2
Krškopolje_con	20.7	35.2	0.0183	116	120	190	854	27.1	12.8
Krškopolje_eco	23.2	30.7	0.0119	113	122	194	834	27.0	12.4
Mangalitsa	23.1	28.4	0.0101	50	206	323	515	26.0	6.2
Moravka	22.8	31.1	0.0087	54	173	282	550	20.2	6.6

^1^ NE_50_–NE_100_: net daily energy intake at 50 and 100 kg BW; ADG: average daily gain; ADFI: average daily feed intake; PDm: average protein deposition; SID Lys. req.: average standardized ileal digestible lysine requirement. ^2^ The numbers (1, 2, 2a, 2b) or indications (conv: conventional; alter: alternative; con: conventional; eco: ecological) following the names of the breeds refer to different studies (see details in the text and in Appendix A).

## Appendix A

For all the tables, nutritional composition is shown on an as-fed basis. The net energy (NE) content is calculated from analyzed composition, if available.

**Table A1:** Data on Alentejana breed used for modelling [10] ^1^.

Trial 1				Trial 2			
Weight, kg	No. of Pigs	Days on Trial	Feed Intake kg/Pig/d	Weight, kg	No. of Pigs	Days on Trial	Feed Intake kg/Pig/d
41.5–54.2	22	28	2.50	35.3–45.8	39	28	1.83
54.2–69.6	22	28	3.15	45.8–59.7	39	30	2.08
69.6–83.1	22	28	3.58	59.2–95.1	13	78	2.82
83.1–96.3	22	34	4.68				

^1^ Diet trial 1: 9.14 MJ NE/kg, 15.7% protein; Diet trial 2: 8.51 MJ NE/kg, 15.2% protein.

**Table A2:** Data on Basque breed used for modelling [11] ^1^.

Group—Conventional				Group—Alternative			
Weight, kg	No. of Pigs	Days on Trial	Feed Intake kg/Pig/d	Weight, kg	No. of Pigs	Days on Trial	Feed Intake kg/Pig/d
34.8–74.7	20	64	2.23	35.3–75.4	20	60	2.43
74.7–109.1	20	70	2.51	75.4–111.5	20	70	2.64
109.1–139.9	20	80	2.41	111.5–146.3	20	76	2.85

^1^ Diet 1: from 35–75 kg, 9.59 MJ NE/kg, 18.0% protein; Diet 2: from 75–145 kg, 8.96 MJ NE/kg, 14.7% protein.

**Table A3:** Data on Bísara breed used for modelling.

[12] ^1^				Project TREASURE ^2^				
Weight, kg	No. of Pigs	Days on Trial	Feed Intake kg/Pig/d	Weight, kg	No. of Pigs	Days on Trial	Feed Intake kg/Pig/d	
							Feed	Corn
29.8–95.8	24	118	2.052	25.3–34.3	10	14	1.5	0
95.8–144.3	24	91	2.618	34.3–42.9	10	14	1.5	0
				42.9–52.4	10	14	1.5	0.3
				52.4–59.4	10	14	1.5	0.3
				59.4–68.8	10	14	1.5	0.4
				68.8–74.6	10	14	1.5	0.4
				74.6–80.5	10	14	1.5	0.4
				80.5-–90.1	10	14	1.5	0.6
				90.1–96.7	10	14	1.5	0.6
				96.7–103.4	10	14	1.5	0.6
				103.4–110.6	10	14	1.5	0.6
				110.6–115.8	10	14	1.5	0.6

^1^ Diet: 9.51 MJ NE/kg, 15.0% protein; ^2^ TREASURE study: Diet 1 for 28 days, 9.75 MJ NE/kg, 16.7% protein; Diet 2 for 140 days, 9.43 MJ NE/kg, 15.3% protein; corn: 9.90 MJ NE/kg, 8.6% protein.

**Table A4:** Data on Apulo Calabrese breed used for modelling [13] ^1^.

Group without soy ^2^				Group with soy ^3^			
Weight, kg	No. of Pigs	Days on Trial	Feed Intake kg/Pig/d	Weight, kg	No. of Pigs	Days on Trial	Feed Intake kg/Pig/d
40.2–72.2	36	42	2.3	33.8–62.7	36	42	2.1
72.2–115.2	36	56	3.3	62.7–105.4	36	56	3.3
115.2–139.1	36	44	3.7	105.4–35.2	36	44	3.5

^1^ Diet 1 app. 30–70 kg; Diet 2 app. 70–130 kg; ^2^ Diet 1: 9.34 MJ NE/kg, 15.4% protein; Diet 2: 9.69 MJ NE/kg, 14.2% protein; ^3^ Diet 1: 9.22 MJ NE/kg, 15.5% protein; Diet 2: 9.70 MJ NE/kg, 14.2% protein.

**Table A5:** Data on Cinta Senese breed used for modelling (Project TREASURE) ^1^.

Weight, kg	No. of Pigs	Days on Trial	Feed Intake kg/Pig/d
38.0–46.45	11	29	1.23
46.45–57.95	11	56	1.82
57.95–67.77	11	84	2.00
67.77–85.27	11	119	2.40
85.27–95.45	11	149	2.65
95.45–106.23	11	183	2.80
106.23–123.9	11	212	3.37
123.9–143.35	11	246	3.32

^1^ Diet 1: from start until 119 days on trial; 9.64 MJ NE/kg, 12.4% protein. Diet 2: from 119 until 246 days on trial; 9.71 MJ NE/kg, 10.3% protein.

**Table A6:** Data on Iberian breed used for modelling [14] ^1^.

Weight, kg	No. of Pigs	Days on Trial	Feed Intake kg/Pig/d
16.1–35.9	29	68	0.91
35.9–103.2	20	135	2.08
103.2–159.5	20	70	3.28

^1^ Diet 1 before 36 kg: 10.00 MJ NE/kg, 17.8% protein; Diet 2 from 36 to 101 kg: 9.50 MJ NE/kg, 15.8% protein; Diet 3 from 101 to 158 kg live body weight: 10.40 MJ NE/kg, 13.5% protein.

**Table A7:** Data on Iberian breed used for modelling [15].

Reduced Protein Diet in Growing Phase ^1^				Usual Protein Diet in Growing Phase ^2^			
Weight, kg	No. of Pigs	Days on Trial	Feed Intake kg/Pig/d	Weight, kg	No. of Pigs	Days on Trial	Feed Intake kg/Pig/d
15.7–50.8	8	68.3	1.78	16.0–48.5	8	67.4	1.76
51.9–114.0	6	86.8	3.13	48.1–114.0	6	107	3.05

^1^ Diet: 9.23 MJ NE/kg, 12.6% protein; ^2^ Diet: 9.08 MJ NE/kg, 16.6% protein.

**Table A8:** Data on Krškopolje pig breed used for modelling (Project TREASURE) ^1^.

Conventional Rearing System ^2^ Diet 1 (8.52 MJ NE/kg, 15.3% Protein) Diet 2 (8.82 MJ NE/kg, 14.0% Protein)				Ecological Rearing System ^3^ Diet 1 (8.69 MJ NE/kg, 15.0% Protein) Diet 2 (8.83 MJ NE/kg, 14.0% Protein)			
Weight, kg	No. of Pigs	Days on Trial	Feed Intake kg/Pig/d	Weight, kg	No. of Pigs	Days on Trial	Feed Intake kg/Pig/d
36.2–45.4	12	14	1.88	35.0–43.7	12	14	2.22
45.4–56.6	12	14	2.63	43.7–54.1	12	14	2.64
56.6–69.4	12	14	2.99	54.1–66.4	12	14	2.98
69.4–83.2	12	14	3.56	66.4–79.2	12	14	3.54
83.2–94.3	12	14	3.26	79.2–91.7	12	14	3.26
94.3–107.3	12	13	3.26	91.7–103.5	12	13	3.26
107.3–111.8	12	6	3.26	103.5–115.4	12	6	3.35
111.8–116.4	12	12	3.45	115.4–117.5	12	12	3.35
116.4–120.4	12	14	3.45	117.5–124.3	12	14	3.35

^1^ Diet 1 was used for the first 28 days; ^2^ Diet 1: 8.52 MJ NE/kg, 15.3% protein; Diet 2: 8.82 MJ NE/kg, 14.0% protein; ^3^ Diet 1: 8.69 MJ NE/kg, 15.0% protein; Diet 2: 8.83 MJ NE/kg, 14.0% protein.

**Table A9:** Data on Mangalitsa (Swallow-Belly type) and Moravka breeds used for modelling (Project TREASURE) ^1^.

Mangalitsa				Moravka			
Weight, kg	No. of Pigs	Days on Trial	Feed Intake kg/Pig/d	Weight, kg	No. of Pigs	Days on Trial	Feed Intake kg/Pig/d
24.3–35.9	12	35	1.43	29.9–46.2	10	35	1.61
35.9–44.8	12	19	1.97	46.2–55.6	10	19	2.24
44.8–59.2	12	28	2.5	55.6–71.5	10	28	2.74
59.2–79.2	12	29	2.67	71.5–89.1	10	29	3.10
79.2–97.0	12	30	3.08	89.1–103.6	10	30	3.50
97.0–104.1	12	31	2.58	103.6–118.9	10	31	2.82
104.1–115.5	12	26	2.79	118.9–131.3	10	26	2.97

^1^ Diet 1 was used for the first 82 days of trial, 9.98 MJ NE/kg, 13.7% protein; Diet 2 was used for the last 116 days of trial, 10.00 MJ NE/kg, 14.4% protein.

## References

[1] Emmans G.C., Kyriazakis I. (1997). Models of pig growth: Problems and proposed solutions. Livest. Prod. Sci..

[2] Van Milgen J., Noblet J., Dourmad J.Y., Labussière E., Garcia-Launay F., Brossard L. (2012). Precision pork production: Predicting the impact of nutritional strategies on carcass quality. Meat Sci..

[3] Van Milgen J., Valancogne A., Dubois S., Dourmad J.Y., Sève B., Noblet J. (2008). InraPorc: A model and decision support tool for the nutrition of growing pigs. Anim. Feed Sci. Technol..

[4] National Research Council (2012). Nutrient Requirements of Swine.

[5] Fernández-Fígares I., Lachica M., Nieto R., Rivera-Ferre M.G., Aguilera J.F. (2007). Serum profile of metabolites and hormones in obese (Iberian) and lean (Landrace) growing gilts fed balanced or lysine deficient diets. Livest. Sci..

[6] Barea R., Nieto R., Aguilera J.F. (2007). Effects of the dietary protein content and the feeding level on protein and energy metabolism in Iberian pigs growing from 50 to 100 kg body weight. Animal.

[7] Nieto R., Lara L., Barea R., García-Valverde R., Aguinaga M.A., Conde-Aguilera J.A., Aguilera J.F. (2012). Response analysis of the Iberian pig growing from birth to 150 kg body weight to changes in protein and energy supply. J. Anim. Sci..

[8] Vautier B., Quiniou N., van Milgen J., Brossard L. (2013). Accounting for variability among individual pigs in deterministic growth models. Animal.

[9] Čandek-Potokar M., Nieto R. (2019). European Local Pig Breeds–Diversity and Performance. A Study of Project Treasure.

[10] Freitas A.B. (1998). Influência do Nível e Regime Alimentar em Pré-Acabamento Sobre o Crescimento e Desenvolvimento do porco Alentejano e suas Repercussões Sobre o Acabamento em Montanheira e com Alimento Comercial. Ph.D. Thesis.

[11] Lebret B., Dourmad J.Y., Mourot J., Pollet P.Y., Gondret F. (2014). Production performance, carcass composition, and adipose tissue traits of heavy pigs: Influence of breed and production system. J. Anim. Sci..

[12] Santos e Silva J., Ferreira-Cardoso J., Bernardo A., Pires da Costa J.S., Wenk C., Fernandez J.A., Dupuis M. (2000). Conservation and development of the Bísaro pig. Characterisation and zootechnical evaluation of the breed for production and genetic management. Proceedings of the Joint Session of the EAAP Commissions on Pig Production, Animal Genetics and Animal Nutrition.

[13] Rossi A., Ferrari P., Bossio M.B., Moncao F., Fusaro A. Growth performance and meat quality of outdoor reared Calabrese pigs. Proceedings of the 6th International Symposium on the Mediterranean Pig.

[14] Ayuso M., Óvilo C., Fernández A., Nunez Y., Isabel B., Dazac A., López-Bote C.J., Rey A.I. (2015). Effects of dietary vitamin A supplementation or restriction and its timing on retinol and α-tocopherol accumulation and gene expression in heavy pigs. Anim. Feed Sci. Technol..

[15] Barea R., Nieto R., Vitari F., Domeneghini C., Aguilera J.F. (2011). Effects of pig genotype (Iberian v. Landrace x Large White) on nutrient digestibility, relative organ weight and small intestine structure at two stages of growth. Animal.

[16] Nieto R., Miranda A., García M.A., Aguilera J.F. (2002). The effect of dietary protein content and feeding level on the rate of protein deposition and energy utilization in growing Iberian pigs from 15 to 50 kg body weight. Br. J. Nutr..

[17] Čandek-Potokar M., Batorek Lukač N., Tomažin U., Nieto R. Performances de croissance des races locales de porcs selon la phase de production: Une étude analytique du projet TRESAURE. Proceedings of the 51èmes Journées de la Recherche Porcine.

[18] Čandek-Potokar M., Batorek Lukač N., Tomažin U., Škrlep M., Nieto R., Čandek Potokar M., Nieto R. (2019). Analytical review of productive performance of local pig breeds. European Local Pig Breeds–Diversity and Performance. A Study of Project Treasure.

